# One‐Step Nucleic Acid Amplification Analysis of Sentinel Nodes in Endometrial Cancer Versus Ultrastaging: First Long‐Term Follow‐Up Data of Discordant Cases

**DOI:** 10.1002/cnr2.70082

**Published:** 2024-12-26

**Authors:** Jan Kosťun, Khaled M. Ismail, Martin Pešta, Robert Slunečko, Petr Stráník, Vendula Smoligová, Jiří Presl

**Affiliations:** ^1^ Department of Gynaecology and Obstetrics, University Hospital Pilsen and Faculty of Medicine in Pilsen Charles University Plzeň‐Lochotín Czech Republic; ^2^ Department of Gynaecology and Obstetrics and Biomedical Center, University Hospital Pilsen and Faculty of Medicine in Pilsen Charles University Plzeň‐Lochotín Czech Republic; ^3^ Department of Immunochemistry, University Hospital Pilsen and Faculty of Medicine in Pilsen Charles University Plzeň‐ Bory Czech Republic; ^4^ Šikl's Department of Pathology, University Hospital Pilsen and Faculty of Medicine in Pilsen Charles University Plzeň‐Lochotín Czech Republic

**Keywords:** endometrial cancer, follow‐up, OSNA, sentinel node

## Abstract

**Aims:**

Endometrial cancer (EC) is the most common gynecological cancer worldwide and its incidence is rising. The cornerstone of its management is surgical treatment with nodal staging. A monocentric study investigating the potential of the molecular biology method of one‐step nucleic acid amplification (OSNA) in sentinel lymph node (SLN) analysis was conducted at our institution between April 2016 and January 2018. Histopathological ultrastaging was used as the reference standard for SLN examination and OSNA as the index test. The aim of this study was to assess the long‐term outcome of patients with discordant SLN and OSNA results. To our knowledge, this is the first study exploring this issue.

**Methods and Results:**

Patients were followed in line with the current ESMO/ESGO/ESTRO recommendations. The institutional electronic database was retrospectively searched for patients' follow‐up data from April 2016 till March 2023. Only patients who provided a written valid consent and had a positive OSNA and negative ultrastaging of their SLN analysis were included in the study. The primary endpoint was the retrospective analysis of their clinical outcome. Data from 58 patients enrolled into our previous study were reviewed and 12 discordant patients who met the inclusion criteria for this study were identified. The median follow‐up was 83 months. Disease recurrence was detected in 3 (25%) patients, two of these were nodal and both patients died. One patient had a solitary lung metastasis which was surgically treated, and the patient was disease‐free during the whole study period.

**Conclusion:**

The recurrence rate of patients included in the study was in the intermediate‐high and high‐risk group range, and hence, higher than expected based on ultrastaging results. Furthermore, benign epithelial inclusions do not seem to adversely affect OSNA SLN analysis in EC patients.

## 1 Introduction

The current gold standard in sentinel lymph node (SLN) analysis in endometrial cancer (EC) is histopathological ultrastaging with utilization of immunohistochemistry. It is used to detect low‐volume metastases in patients with apparent uterine‐confined EC. It is a widely accepted diagnostic approach and a crucial component of the patient management algorithm [1]. Intracervical tracer injection, first superficially (1–3 mm) and then deeper into the cervical stroma (1–2 cm), has become the established node detection technique [2, 3]. This enables the dye to reach lymphatic channels responsible for both the uterine corpus and the cervix. This method has proven to be more effective and practical for clinical use in contrast to hysteroscopic or intracorporeal tracer dye application. Indocyanine green (ICG) is currently the tracer of choice, displacing patent blue alone or its combination with technetium [4, 5].

This detailed analysis allows the detection of low‐volume micrometastatic disease (ranging from > 0.2 to 2 mm) or even isolated tumor cells (≤ 0.2 mm). Nonetheless, there is a lack of universal standardization in the ultrastaging technique. Furthermore, the required time for analysis (2 weeks in general) and pathologist's expertise are additional limitations to ultrastaging.

One‐step nucleic acid amplification (OSNA) has been proposed as an alternative methodology for sentinel node assessment. The methodology is standardized, automated, and fast, with results available within 20–30 min. OSNA relies on the detection of cytokeratin 19 mRNA in lymphatic tissue. The method was first introduced in 2006, gradually gained popularity, and is currently an established diagnostic test in the management of some malignant diseases, such as breast or colorectal cancer [6, 7, 8, 9]. In the field of EC, however, there are still insufficient robust data to introduce this method into routine clinical practice; hence, the method remains experimental.

The performance of the OSNA method was investigated at our institution in the period between April 2016 and January 2018. SLNs of patients with EC were analyzed using both OSNA (index test) and histopathological ultrastaging (reference test). Some patients provided discordant results. However, further clinical management was based on the ultrastaging results. Their subsequent treatment and follow‐up were carried out in line with current internationally agreed evidence‐based guidelines [10].

The aim of this study was to focus on patients who had discordance in their SLN examination with OSNA positive and ultrastaging generating negative results, and retrospectively analyze their clinical outcomes. According to data published so far, 10%–23% of patients with EC will suffer a disease recurrence [11, 12]. The overall recurrence rate, among all stages of the disease, is about 20% in endometrioid histology and up to 50% in other histological types [13]. We hypothesized that our group of patients would have a higher proportion of nodal recurrences (according to the OSNA positive result) than patients without nodal involvement (according to the negative ultrastaging result), i.e. in early‐stage disease, where the recurrence rate is expected to be around 10% [14, 15].

## 2 Patients and Methods

This was a monocentric retrospective long‐term follow‐up study, which was conducted at our institution from April 2016 to March 2023. Patients > 18 years who participated in our previous study analyzing the clinical use of OSNA in sentinel node examination in EC [16] and had discordance in the results of at least one SLN analysis where OSNA demonstrated positivity while ultra staging was negative were included in this study. Patient informed consent for follow‐up was already part of a pilot project dealing with sentinel node analysis in EC with OSNA. Patients who did not fulfill all the above criteria were excluded from this analysis.

SLNs were processed in the pilot study as follows: nodes larger than 5 mm were cut perpendicularly to the longitudinal axis into 2 mm slices, and nodes 5 mm and smaller were halved longitudinally. Odd sections were analyzed by OSNA, while even sections were by ultrastaging. The nature of both methods precluded the examination of the sample by both techniques.

All patients were managed in line with the current ESMO/ESGO/ESTRO recommendations based on their FIGO staging which relied on the results of sentinel node immunohistochemical ultrastaging [10]. The 2009 FIGO classification, most up‐to‐date at the time of the pilot study, was used for staging and management [17]. Follow‐up data were collected from our hospital electronic records and analyzed focusing on disease recurrence, disease‐free interval, and overall survival as long‐term core clinical outcomes.

All subjects gave their informed consent for inclusion before they participated in the study. The study was conducted in accordance with the Declaration of Helsinki, and the protocol was approved by the Local Ethics Committee of Faculty Hospital in Pilsen, Czech Republic (https://okf.fnplzen.cz/cs/node/1536).

## 3 Results

We identified 12 patients meeting the inclusion criteria. Their characteristics are summarized in Table 1. Of these, three suffered recurrence during the follow‐up period.

**TABLE 1:** Patients characteristics at baseline and follow‐up.

Patient Nr.	Age at diagnosis	FIGO according to OSNA	FIGO according to histology	Benign epithelial inclusions in SLN	Follow‐up in months	Recurrence of the disease during follow‐up	Status to 9/2023
1.	74	IIIC1	IA	No	112	No	Alive
2.	71	IIIC1	II	No	108	Yes	Alive
3.	72	IIIC1	IA	No	108	No	Alive
4.	30	IIIC1	IA	No	108	No	Alive
5.	69	IIIC1	II	No	21	Yes	Deceased
6.	66	IIIC1	II	No	54	Yes	Deceased
7.	51	IIIC1	IA	Yes	91	No	Alive
8.	61	IIIC1	IA	No	90	No	Alive
9.	51	IIIC1	IA	No	76	No	Alive
10.	60	IIIC1	IB	No	75	No	Alive
11.	55	IIIC1	IA	No	74	No	Alive
12.	56	IIIC1	IA	No	67	No	Alive

Their characteristics are detailed in Table 2. The median follow‐up duration of our study was 83 months (range of 21–112 months, interquartile range 37.5, standard deviation 26.89).

**TABLE 2:** Characteristics of patients with disease recurrence.

	Patient Nr. 2	Patient Nr. 5	Patient Nr. 6
Age at diagnosis	71	69	66
Primary tumour size, greatest diameter in mm	55	55	32
Primary tumor histology, grade	Endometrioid, G2	Clear cell, G3	Endometrioid, G2
LVSI	Yes	Yes	No
Molecular classification	Mismatch repair‐deficient tumour—MMRd	Nonspecific molecular profile—NSMP	Nonspecific molecular profile—NSMP
Node 1 ultrastaging	Negative	Negative	Negative
Node 2 ultrastaging	Negative	Negative	Negative
Node 1 CK‐19 copy number μ/L	270	410	35 000
Node 1 OSNA result	Micrometastases	Micrometastases	Macrometastases
Node 2 CK‐19 copy number μ/L	620	270	3800
Node 2 OSNA result	Micrometastases	Micrometastases	Micrometastases
Maximal discordance micrometastases	Yes	Yes	No
Maximal discordance macrometastases	No	No	Yes
FIGO according to histology	II	II	II
FIGO according to OSNA	IIIC1	IIIC1	IIIC1
Adjuvant therapy	BRT* + EBRT**	BRT + EBRT	BRT + EBRT
Time to recurrence in months	53	5	42
Site of recurrence	Lung, solitary metastasis	Retroperitoneal lymphatic nodes, abdominal wall	Retroperitoneal and inguinal lymphatic nodes
Recurrence therapy	Surgical excision	Palliative CHT	Surgical excision
Status to 9/2023	Alive	Deceased	Deceased

* Brachytherapy.

** External beam radiotherapy.

In one of these patients, disease recurrence was observed after 53 months as a solitary pulmonary metastasis. This patient was initially classified as FIGO stage II endometroid carcinoma G2, LVSI+ and with an intermediate risk molecular profile of MMRd. The patient subsequently underwent adjuvant radiotherapy. After 53 months of follow‐up, solitary pulmonary metastasis was detected and surgically removed. The patient is currently being followed‐up with no further evidence of recurrence.

The second patient who had a recurrence had an aggressive histological type of endometrial clear cell carcinoma, G3 with positive LVSI, and nonspecific molecular profile. The patient was classified as FIGO II according to histological ultrastaging and adjuvant radiotherapy was indicated. Within 5 months (while still under radiotherapy because the start of the therapy was postponed at the patient's request) the disease generalized to the retroperitoneal lymph nodes and abdominal wall. Therefore, palliative chemotherapy was indicated. The patient sadly died after seven cycles.

The third patient with recurrence was a 66‐year‐old female with endometroid carcinoma, G2, and nonspecific molecular profile. Based on histopathology, the patient was classified as FIGO stage II. The patient subsequently underwent adjuvant radiotherapy. At 42 months postdiagnosis, the disease relapsed with involvement of the retroperitoneal nodes. Due to the favorable paraaortic position of the recurrence, surgical excision was performed. However, generalization to other paraaortic and inguinal lymphatic nodes followed, and the patient died in 10 months. (Table 2).

In our retrospective review, we also identified one case of downstaging by OSNA of an 84‐year‐old staged as IIIC1 by ultrastaging and IB according to OSNA. This patient was diagnosed in April 2017 and had radiotherapy following her surgery (the recommended chemotherapy was rejected by the patient) and was disease‐free at follow‐up at the time of data collection for this study.

## 4 Discussion

Metastatic lymph node involvement is a major prognostic factor and is associated with a worsening of 5‐year overall survival to 44%–52%. Even in the case of disease limited to the uterine body, the risk of micrometastases is 5%–18% [18].

Our group previously published the results of a monocentric pilot study, which was the first to compare the diagnostic accuracy of OSNA (index test) to histopathological ultrastaging (reference standard) for SLN examination in patients with EC. Of the 58 patients included in the study, a total of 135 sentinel nodes were examined, the results of both diagnostic methods were concordant in 116 cases, thus achieving a sensitivity, specificity, and diagnostic accuracy of 90.9%, 85.5%, and 85.9% respectively. However, there were discrepancies in the OSNA versus ultrastaging‐based FIGO staging in more than 20% of patients (FIGO 2009). Indeed, some patients classified as FIGO stages I and II would have been classified as FIGO stage IIIC1 according to OSNA [16]. Nevertheless, the clinical management of all patients in this study was based on the results of ultrastaging (the reference standard). It is important to stress that tissue examined by OSNA cannot be analyzed histologically and vice versa. In this previous OSNA performance pilot study, the nodal tissue was sliced (2 mm thick slices) perpendicular to its longitudinal axis. Odd and even number slices were then allocated to OSNA or ultrastaging assessments respectively. Given the method of lymph node slicing, it is plausible that sample allocation could have biased the results, particularly in cases where a micrometastases was identified as the involvement could be < 2 mm.

However, similar results demonstrating the diagnostic capabilities of OSNA were presented in a meta‐analysis by Raffone et al. in 2020. This work described the potential of OSNA in detecting lymph node metastases in EC compared with histopathological examination where data from 4 studies, 237 patients and 691 lymph nodes were included. OSNA achieved an overall sensitivity of 88%, a specificity of 93%, and a diagnostic accuracy of 95.9% compared to ultrastaging [19]. However, Raffone and colleagues did not provide any follow‐up data.

Despite previous reports relating to the use of OSNA in EC, to our knowledge, our study is the first to present long‐term follow‐up data for EC patients staged based on their SLN assessment using OSNA [20, 21].

Patients included in this study were treated based on the histological result of their SLN ultrastaging. Patients with recurrence were in all cases FIGO stage II (FIGO 2009). All these patients received postoperative radiotherapy. In the case of classification as FIGO stage IIIC1 according to positive OSNA results of the nodes, chemotherapy would have been indicated along with radiotherapy [22]. It is possible that this could have had led to higher postoperative disease control, especially in the patient with pulmonary metastasis.

Patients at low and intermediate risk are associated with a risk of disease recurrence of around 9%–16%, and this group would include the studied patients if classified according to ultrastaging. Nevertheless, the recurrence rate in our patients was 25%. The recurrence rate is reported to be 17%–40% in intermediate‐high and high‐risk patients [23, 24]. This level of recurrence would correspond better to groups of intermediate‐high and high‐risk patients. Indeed, these patients would have been in this category if they were classified according to OSNA results in FIGO IIIC1.

All three patients with disease recurrence were initially staged as FIGO II and received adjuvant brachytherapy (BRT) and external beam radiation therapy (EBRT). However, in two of them, it did not prevent the locoregional recurrence. Radiation therapy is the most recommended tool to prevent locoregional disease recurrence and the standard treatment regimen for locoregional recurrences in patients who have not had radiation therapy before. According to a meta‐analysis published in 2023 by Ronsini et al. [23], which included 15 previously published papers and data from 3205 patients, radiotherapy is the most used therapeutic modality for this purpose. Nevertheless, in our case, surgical excision of recurrence was indicated in two of our patients. The patient with solitary pulmonary metastasis benefited from this approach. However, the patient with paraaortic recurrence died of generalized disease.

In all three patients with recurrences, histological examination of the nodes was completely negative. OSNA detected micrometastases in two patients and a macrometastasis in the third patient (Table 2). The majority of patients included in our study group were discordant for micrometastases; however, the management approach to micrometastases is still controversial. Although adjuvant radiotherapy and chemotherapy are often recommended, not all studies fully support this. Ronsini et al. published a meta‐analysis in 2024 containing data from 1682 patients with FIGO I‐IV EC with the aim of assessing the effect of radiotherapy and chemotherapy on the outcome of patients with nodal micrometastases. However, the administration of adjuvant therapy in these patients did not appear to be associated with a statistically significant improved survival outcome [25].

Benign epithelial inclusions in sentinel node were histologically confirmed in only one case in an OSNA positive (710 copies/μL of mRNA CK‐19) and ultrastaging negative patient (Table 1, patient Nr. 7). Adjuvant treatment was not administered, and the patient did not show disease recurrence during the follow‐up period. In this case, the eventual administration of adjuvant therapy based solely on the OSNA positive result would have resulted in overtreatment. Benign epithelial inclusion such as endo‐salpingiosis can be detected in SLNs in around 2% and in non‐SLNs in 0.01%–0.02% of cases [20, 26, 27]. This phenomenon can lead to false OSNA positivity of the examined nodes. However, in our opinion, such a low incidence should not have a statistically significant effect on overall outcomes and, hence, is not a limitation to the use of the OSNA analysis in a clinical setting.

We recognize that our small sample size is a limitation to this analysis; however, we wanted to explore the long‐term outcomes of this well‐defined cohort of patients with discordance between their OSNA and ultrastaging‐based tumor staging.

Another limitation, which is inherent to the diagnostic modalities used, is the inability to subject the same tissue sample to both OSNA and ultrastaging analysis. The nature of both methods hinders this which could have introduced allocation bias. This issue can be mitigated by random allocation of a much larger sample size. Finally, in this study, an OSNA RD 100i analyzer and the corresponding detection kit were used—originally developed for node analysis in breast carcinoma. A new generation of the OSNA RD 210 analyzer and the LYNOAMP CK19E detection kit have been developed since. This set has been adapted specifically for patients with EC. Differences in sensitivity and specificity between the two generations of equipment are to be expected. Nonetheless, the novelty of our research question, our strict inclusion criteria and diagnostic methodologies used, and the potential impact of our work on reducing tumor recurrence are major strengths of our work.

## 5 Conclusion

This study is the first to report long‐term follow‐up data on EC patients who underwent OSNA sentinel node analysis. We focused on patients who had discordant sentinel node involvement in terms of OSNA‐positive and ultrastaging negative results. The overall incidence of recurrence in this study group was higher than expected according to ultrastaging results. The recurrence rate (25%) of patients included in the study would have been expected if patients were in the intermediate‐high and high‐risk group range. This would have been the case if these patients were classified according to the OSNA method. Moreover, benign epithelial inclusions do not seem to have a significant negative impact on the use of OSNA in SLN analysis in patients with EC. However, in view of the limited sample size, our findings should be interpreted with caution till further evidence is available from larger multicenter studies.

## Author Contributions

**Jan Kosťun:** conceptualization (equal), investigation (equal), writing – original draft (equal). **Khaled M. Ismail:** writing – review and editing (equal). **Martin Pešta:** investigation (equal), methodology (equal). **Robert Slunečko:** investigation (equal), methodology (equal). **Petr Stráník:** investigation (equal). **Vendula Smoligová:** investigation (equal). **Jiří Presl:** conceptualization (equal), supervision (equal).

## Consent

As part of their informed consent for the pilot study, all patients gave their informed consent for the publication of their follow‐up data.

## Conflicts of Interest

The authors declare no conflicts of interest.

## Data Availability

The data that support the findings of this study are available from the corresponding author upon reasonable request.
